# Increased pollinator service and reduced pollen limitation in the fixed dune populations of a desert shrub

**DOI:** 10.1038/s41598-017-17253-w

**Published:** 2017-12-04

**Authors:** Cheng-Chen Pan, Hao Qu, Qi Feng, Lin-De Liu, Ha-Lin Zhao, Yu-Lin Li, Yu-Qiang Li, Tong-Hui Zhang, Xin-Ping Liu

**Affiliations:** 1 Northwest Institute of Eco-Environment and Resources, Chinese Academy of Sciences, Lanzhou, 730000 China; 2 Key Laboratory of Ecohydrology of Inland River Basin, Chinese Academy of Sciences, Lanzhou, 730000 China; 3 College of Life Sciences, Ludong University, Yantai, 264025 China

## Abstract

Evaluations of restoration success usually focus on the structural aspects of ecosystems. Pollination, as an important functional aspect, is often overlooked. Here, the shifts in pollinator assemblage and pollen limitation in the desert shrub *Caragana microphylla* were examined along a restoration gradient in Horqin Sand Land, northern China. We identified seven species of bees; however, only four bee species were found to be effective pollinators, with *Xanthosaurus remota* dominating in the fixed dunes, and with no bee species or only a single species, *X. remota*, being observed in the semi-fixed and mobile dunes. Flower visitation rate was nearly ten times higher in the fixed dunes than in the mobile and semi-fixed dunes. Experimental floral manipulations revealed that the fixed dune populations experienced less pollen limitation, along with the increase in pollinator availability. Between the mobile and semi-fixed dune populations, pollen limitation was severe and at similar levels. The intensity of pollen limitation was negatively related to pollinator abundance and richness. Overall, the dependence on pollinators for reproduction may be an important constraint that limits persistence in this system. Increased pollinator service during the restoration process may ameliorate pollen limitation, benefiting the restoration of vegetation in this semiarid sandy area.

## Introduction

Various programs, such as grazing exclusion and planting native species, have been implemented globally to restore degraded lands^1^. Currently, most studies evaluating restoration efforts focus on the restored habitat structure, such as soil properties or the diversity, composition and structure of vegetation and fauna^2–5^. However, changes in the ecosystem structure and function may not display similar trends to those observed in the studied properties^6,7^. Thus, additional empirical studies are required to determine the effects of habitat restoration on the ecosystem structure and function to evaluate the success of restoration projects. These studies will provide a firm foundation for establishing conservation guidelines in degraded regions, such as those in Horqin Sand Land of Inner Mongolia, northern China.

Pollination, as an important ecosystem function, is often overlooked, even though it is essential to the establishment and persistence of plants. Almost 90% of flowering plant species rely on animal pollinators for pollination^8^. This important mutualism can be negatively affected by anthropogenic habitat degradation and loss^9,10^. Recent research on the effects of habitat restoration has shown that this process enhances pollinator communities^11–13^. Studies on the effect of habitat restoration on plant reproduction have suggested that the pollination or fruit set increased^13,14^, but the studies failed to jointly establish pollination increase and pollen limitation (PL). Morandin & Kremen^12^ demonstrated that restoration promoted pollinator populations and caused the migration of native bees to adjacent fields. Other studies have reported that restoration enhanced fruit set^14^. These ameliorations could be explained by aspects of the resource environment in restored habitats that benefit reproductive output via increased pollination rates. Under these circumstances, restoration usually leads to an increase in the number and size of many wild plant and animal populations. However, restoration has been reported to either increase or decrease the abundance of pollinators at different restoration stages^13^. Increase in pollinator populations in restored habitats can significantly increase their services^11^, reduce pollination limitation and consequently, positively affect plant reproductive success^14^. Therefore, a quantitative assessment of ecosystem functions, such as pollination, can expand the understanding of optimal habitat restoration methods and can influence the expectations and goals of stakeholders^15^.

Horqin Sand Land is one of the most severely desertified regions in northern China because of over-grazing and over-cultivation^16^. Since the 1970s, restoration projects, such as grazing exclusion, the construction of physical sand barriers and corn straw fence belts and planting native species, have been undertaken to stabilize dunes and to restore the vegetation around mobile dunes with less than 30% vegetation cover^17^. With these measures in place, mobile dunes have recovered gradually into semi-fixed dunes with 30–60% vegetation cover or fixed dunes with more than 60% vegetation cover^18^. Research over the past three decades on restoration efforts has focused on degraded vegetation and soil^4^. However, the effects on plant pollination have received limited attention during the long-standing process of habitat restoration.

In this study, we investigated pollination of *Caragana microphylla* Lam., an insect-pollinated shrub, which is a primary plant of Horqin Sand Land in northern China. The shrub is widely planted to control land desertification in northern China because of its high drought tolerance, antiwind erosion properties, and N_2_-fixation capacity^19^. However, individual shrub will die in a few years without flat stubble, and thus may affect seed production and long term population viability. We evaluated pollinator assemblages and pollen limitation in *C. microphylla* populations during different restoration stages. Because fruit set and seed production may depend on biotic factors and resources, we performed a pollen supplementation experiment to determine the extent of the effect of pollen limitation on fruit set and seed production^20^. Therefore, we aimed to answer the following questions: (1) Do pollinator assemblage and visitation rate continuously increase following vegetation restoration of mobile dunes? (2) Does pollen limitation vary with vegetation restoration of mobile dunes? (3) Is there any relationship between bee assemblages and pollen limitation during vegetation restoration?

## Results

### Pollinator assemblage and visitation rate following vegetation restoration of mobile dunes

**Figure 1:**
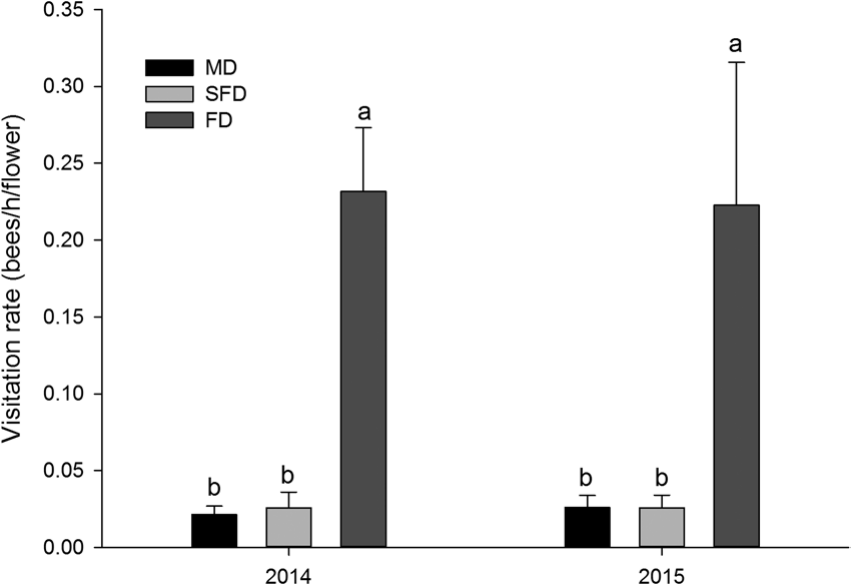
Bee pollinator visitation rate (bees/flower/h) to *Caragana microphylla* in different restoration stages during 2014 and 2015. Values (±SE) are the means of all sites for the mobile dunes (MD), semi-fixed dunes (SFD) and fixed dunes (FD). Different letters indicate significant differences among dune types (*P* < 0.05).

The richness and flower visitation rate of bee species increased significantly from the mobile to the fixed dunes (*P* < 0.05; Fig. 1), but did not greatly differ between years (*P* > 0.05). Over the two flowering seasons, we observed seven species of bees that visited *C. microphylla* flowers in the fixed dune populations (Table 1). Among these species, *Liothyrapis* sp., *Amegilla parhypate* Lidtinek and *Andrenidae* sp. were not pollinators of *C. microphylla* as their flower visits did not involve contact with the anthers or stigmas. *Xanthosaurus remota* Smith, *Megachile desertorum* Morawitz, *Proxylocopa* sp. and *Bombus* sp. were identified as possible pollinators. Over the two flowering seasons, *X. remota* accounted for 77.9% of all effective flower visits (Table 1). However, no bee species or only a single species, *X. remota*, was observed in the semi-fixed and mobile dunes. This pattern remained consistent over both years. Significantly higher bee pollinator richness and flower visitation rate were found in the fixed dune populations; no significant difference was observed between the semi-fixed and mobile dune populations (Fig. 1). The bee pollinator visitation rate was, on average, more than ten times higher in the fixed dune than in the semi-fixed and mobile dune populations.

**Table 1:** Mean visitation rate of the bee visitors to *Caragana microphylla* flowers during different restoration stages in 2014 and 2015. MD = mobile dune, SFD = semi-fixed dune, FD = fixed dune.

	2014			2015		
	MD	SFD	FD	MD	SFD	FD
Megachilidae						
*Xanthosaurus remota* (*Megachile*)	0.02	0.03	0.19	0.03	0.03	0.16
*Megachile desertorum* (*Megachile*)			0.03			0.01
*Liothyrapis* sp.(*Coelioxys*)			0.06			0.04
Apidae						
*Proxylocopa* sp. (*Proxylocopa*)			0.01			0.01
*Bombus* sp. (*Bombus*)						0.02
*Amegilla parhypate* (*Amegilla*)			0.02			0.01
Andrenidae						
*Andrena* sp. (*Andrena*)			0.02			0.01

### Pollen limitation following vegetation restoration of mobile dunes

**Figure 2:**
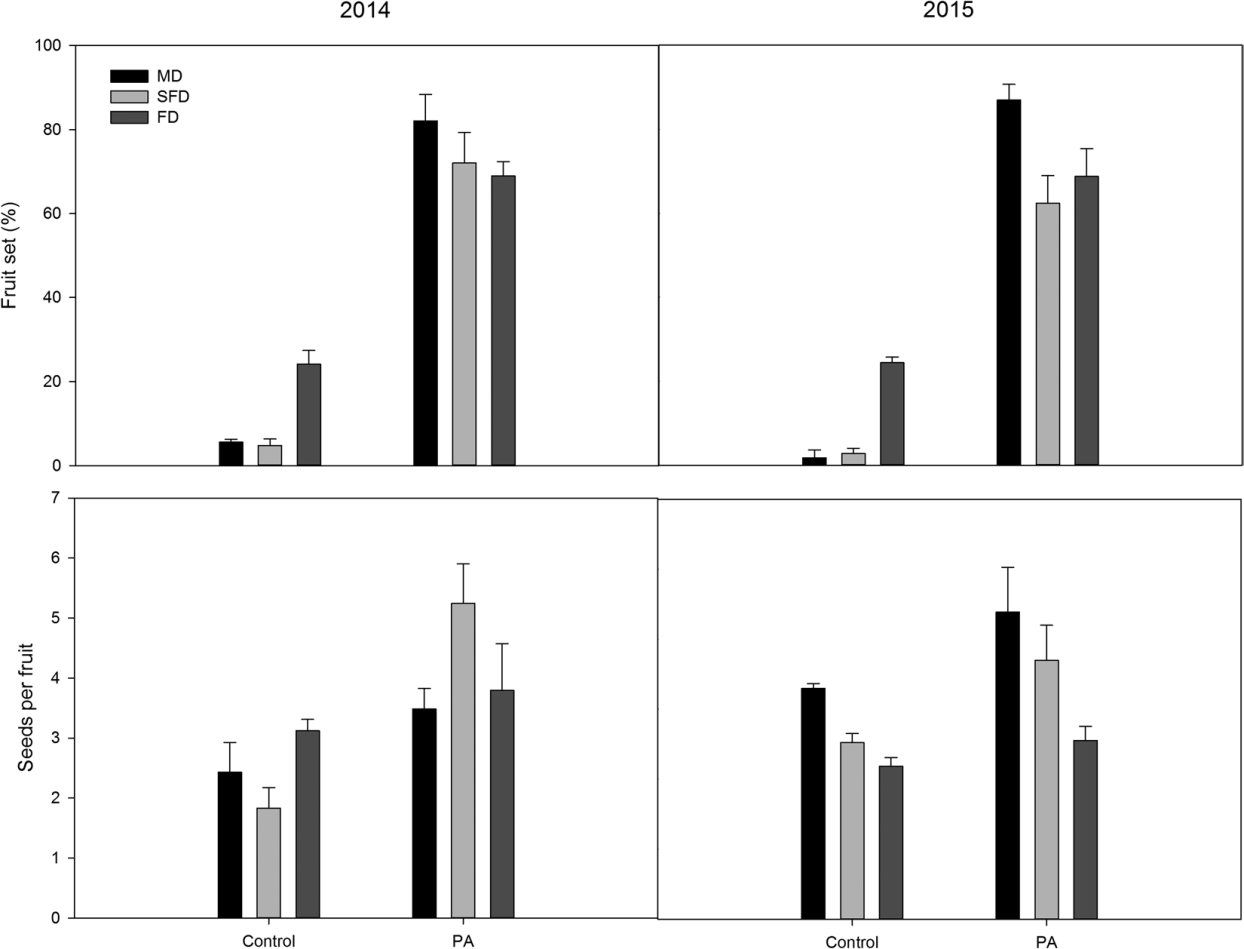
Effect of pollination treatments on the mean fruit set and seeds per fruit of *Caragana microphylla* during different restoration stages in 2014 and 2015. Values (±SE) are the means of all sites for the mobile dunes (MD), semi-fixed dunes (SFD) and fixed dunes (FD). PA = Supplemental outcross treatment.

**Figure 3:**
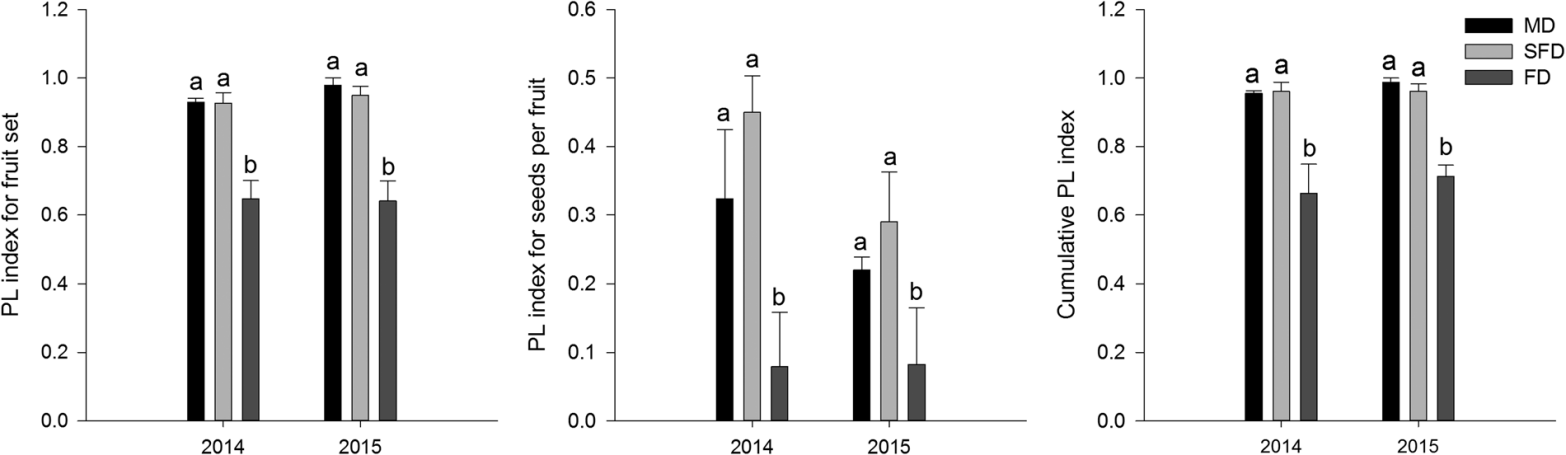
PL index for fruit set, seeds per fruit and cumulative seed production of *Caragana microphylla* during different restoration stages in 2014 and 2015. Values (±SE) are the means of all sites for the mobile dunes (MD), semi-fixed dunes (SFD) and fixed dunes (FD). Different letters indicate significant differences among dune types (*P* < 0.05).

Across all sites combined, the supplemental outcross pollen treatment significantly increased the fruit set and seeds per fruit of the plants compared to the open pollination treatment; this result occurred in both years (Fig. 2), indicating pollen limitation. All 3 PL index values (PL_fruit set_, PL_seeds per fruit_ and PL_cumulative_) were significantly lower in the fixed dune populations during vegetation restoration than in the other studied populations; no significant differences in PL index values were found between the semi-fixed and mobile dune populations (*P* > 0.05; Fig. 3). In addition, no significant effect was observed regarding the year on the variation of PL (*P* > 0.05; Fig. 3). Compared to the mobile dune populations, PL_fruit set_, PL_seeds per fruit_ and PL_cumulative_ decreased by 43.6%, 75.5% and 30.5%, respectively, in 2014 and 34.5%, 62.4% and 27.9%, respectively, in 2015 in the fixed dune populations.

As predicted, PL index was significantly negatively associated with the abundance (PL_fruit set_, *R* ^2^ = 0.34, *P* < 0.01; PL_seeds per fruit_, *R* ^2^ = 0.36, *P* < 0.01; PL_cumulative_, *R* ^2^ = 0.42, *P* < 0.01) and richness of pollinators (PL_fruit set_, *R* ^2^ = 0.53, *P* < 0.01; PL_seeds per fruit_, *R* ^2^ = 0.42, *P* < 0.01; PL_cumulative_, *R* ^2^ = 0.61, *P* < 0.01), i.e., the bee pollinator availability increased and the intensity of pollen limitation decreased substantially in the fixed dunes.

## Discussion

Attempts to restore degraded ecosystems usually focus on the structural aspects of biodiversity^13^. However, the ecosystem structure and function may not change harmonically^7^. Herein, we provide evidence of unparalleled changes in ecosystem structure and function during restoration, as our study showed that over a two-year period, pollinator availability to *C. microphylla* was consistently lower in the mobile and semi-fixed dunes than in the fixed dunes, which resulted in greater pollen limitation of reproduction. In addition, no evidence was found to support autonomous selfing for *C. microphylla* reproduction (data not shown). Therefore, the dependence on pollinators may be an important constraint that limits persistence in this system.

### Pollinator assemblage and visitation rate following vegetation restoration of mobile dunes

The conversion in plant–pollinator interactions from the mobile to the fixed dunes was consistent across both years. Species richness of flower visitors is predicted to increase with restoration^21,22^, but patterns differ among insect groups^23^. In our case, species richness and abundance of bees visiting *C. microphylla* were highest in the fixed dunes, whereas bees were almost entirely absent from the mobile and semi-fixed dunes. This was a surprising result, as it was believed that the restoration of vegetation on the semi-fixed dunes would positively affect the number of bees and flower visits. Species richness of flowering plants has been reported as the best predictor of bee species richness, whereas flowering plant cover best predicts bee abundance^24^. The positive effect of the number of flowering plant species on pollinator species richness and visitation rate may be explained by the increasing floral resource heterogeneity (nectar and pollen), which increases the attractiveness for many pollinator species that forage single and multiple plants^25,26^. At our study sites, plant species richness continuously increased with restoration (Figure S1). However, the level of floral resource utilization by bees was similar between the mobile and semi-fixed dunes, with no difference in the diversity of floral resources (Table S2). In fact, no other floral resource except for *C. microphylla* utilization by bees was found at these sites which may actually prohibit the persistence of pollinators unable to travel between resources^27^. In contrast, the fixed dunes supported continuous floral resources in summer and autumn, creating a wider array of foraging niches for pollinators. Additionally, flower-visiting insects may nest, recolonize, and hibernate in the fixed dunes because many flower-visiting species require suitable locations for hibernation, remaining dormant until spring^28,29^. Therefore, the observed variation in bee community composition (abundance and richness) may be explained by the combined differences in both suitable nesting sites and diverse floral resources, providing a seasonal succession of pollen and nectar resources among habitats. These results suggest that the ability for unparalleled restoration exists among plant richness, vegetation cover and pollinators during the early restoration stage in Horqin Sand Land, northern China. Our results confirm the expectation that more diverse floral resources support more diverse flower-visiting insects^25,30^. The change in flowering plant species richness with restoration was paralleled by peak bee diversity and abundance in the fixed dunes.

### Pollen limitation following vegetation restoration of mobile dunes

Approximately 81% of the studies on the level of pollen limitation investigated a single population during the flowering season^31^. These results showed that reproductive failure due to pollen limitation may lead to a reduction in population growth and long-term viability, resulting in decreases in the abundance of plant species and populations^20,32^. Severe and consistent pollen limitation may even trigger local extinctions in severely degraded habitats^33^. Our study showed a significant variation in pollen limitation across *C. microphylla* populations during the restoration of desertified grasslands. However, pollen limitation intensity of *C. microphylla* appeared to be unaffected by vegetation restoration from the mobile to semi-fixed dunes. A severe pollen limitation was found in the mobile and semi-fixed dune populations (93–99% in 2014 and 2015); therefore, it is clear that the pollen limitation was large enough to threaten population persistence in the mobile and semi-fixed dunes if no evolutionary response occurred. When *C. microphylla* grew on the fixed dunes, reproduction was less limited by pollen (~65% in 2014 and 2015), suggesting that the effect of pollen limitation on population growth is more likely to be lower on the fixed dunes than on the other studied dunes. This finding suggests that fruits are only produced when a sufficient number of pollen grains are delivered to the stigmas. Hence, sexual reproduction contributes to population growth, dynamics and persistence in *C. microphylla* population. The absence of sexual reproduction may result in no population growth at all in this species.

As shown above, variation in vegetation conditions generates variation in the extent to which pollen limits reproductive success in *C. microphylla*, which is consistent with that observed in other terrestrial species^34^. One explanation for this variation in the extent of pollen limitation is the underlying variation in pollinators, such that populations with low pollinator visitation rate and richness suffer from greater pollen limitation than those with high^35,36^. The *C. microphylla* flower visitation rate (bees/h/flower) was more than ten times higher in the fixed dunes than in the semi-fixed and mobile dunes. Given the low pollinator visitation rates recorded in the mobile and semi-fixed dunes and the number of flowers available in the populations, it seems likely that a low fruit set is associated with the number of bees available for pollination. Pollinator diversity is another important factor in enhancing the security of pollination service^37^. Greater pollination success was widely observed in more diverse pollinator communities^36,38–40^. In our study, a negative relationship was found between pollen limitation and pollinator richness in *C. microphylla*. These relationships suggest that increased pollinator availability during vegetation restoration may decrease the intensity of pollen limitation among plants.

Our results demonstrated that vegetation restoration reduced pollen limitation for *C. microphylla* in fixed dunes but not in semi-fixed dunes. This reduction was assumed to result primarily from the restoration effect on floral resources, pollinator diversity and abundance. Animal-mediated processes, such as pollination, are vital to ensure adequate plant reproductive output in restored ecosystems^14^. Our findings also have practical implications for the management of restoration schemes, providing a practical solution for the conservation of flower-visiting insect populations. Future restoration projects should be implemented to enhance floral resources. Habitats that provide a seasonal succession of floral resources can support more diverse pollinators^41,42^. Functional diversity of plant-pollinator interaction enhances the persistence of plant communities^43^. Therefore, increases in floral resources and pollinating insects during the restoration process will be of great value for the survival and propagation of native plant species, which further enhance the restoration process, benefiting the restoration of vegetation, such as in Horqin Sand Land.

## Materials and Methods

### Study species and field site

**Figure 4:**
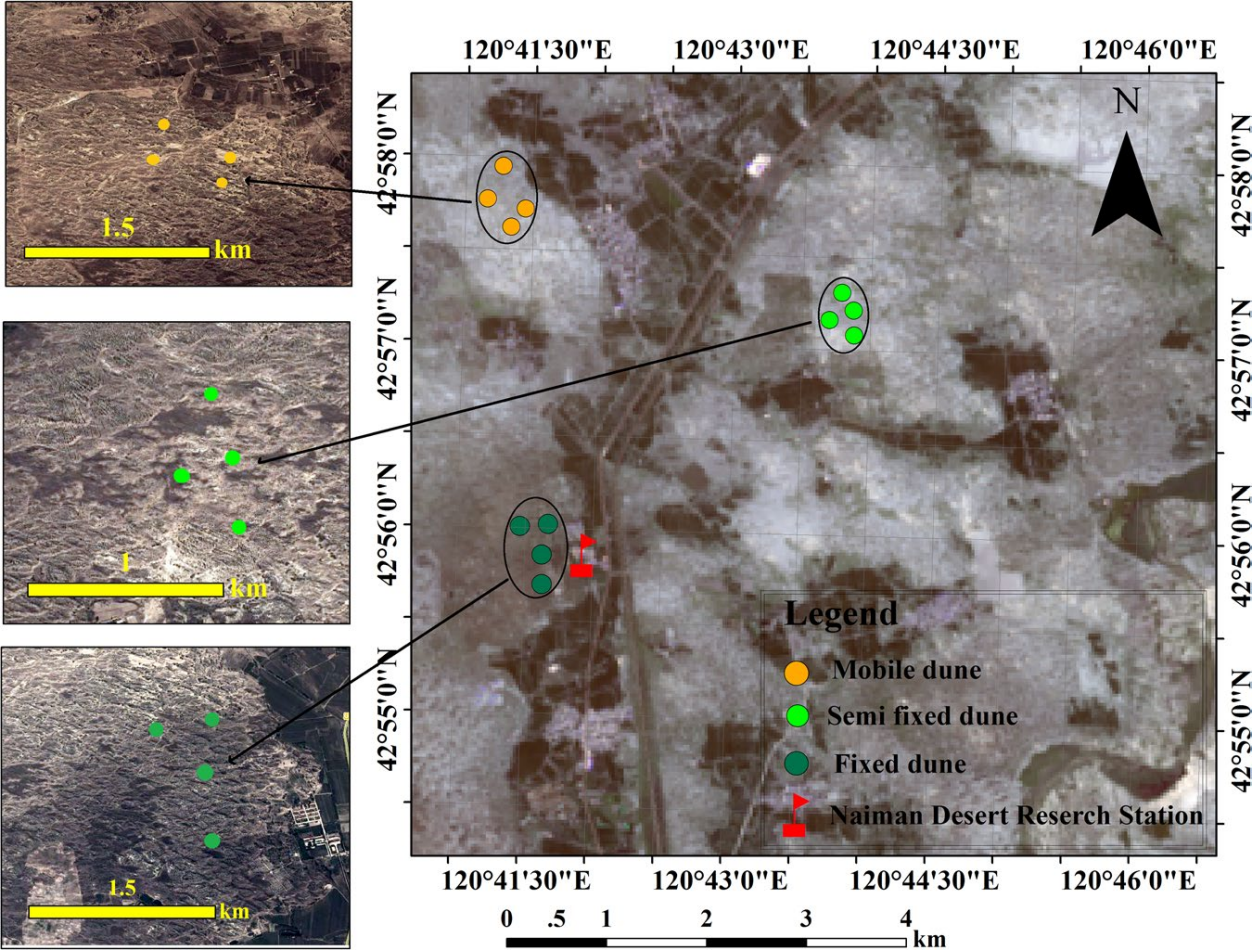
Map showing the location of the three main stages that were studied of the dune stabilization process: mobile dunes, semi-fixed dunes, and fixed dunes in Inner Mongolia, China.

*Caragana microphylla* Lam. (Fabaceae) is a hermaphroditic, perennial shrub and the most dominant of 12 *Caragana* species in the Mongolian steppes^44^. *C. microphylla* grows up to 3 m in height and is a primary plant in the forb-steppe and semi-desert steppe^45^. In northern China, this shrub is widely planted to stabilize mobile dunes from seeds. The shrub blooms massively during May in this region and is the most important floral resource during spring. The yellow flowers of this species are open for 4–5 days, and the pistil and stamens are enclosed in a chamber formed by two ‘keel’ petals. Flower nectaries are situated at the base of the ‘wing’ petals. *C. microphylla* is self-compatible, but pollinators are essential for successful fruit set (data not shown). The study area was located at the Naiman Desertification Research Station of the Chinese Academy of Sciences (42°55′5″N, 120°41′49″E, 371 m a.s.l.) at the southwestern end of Horqin Sand Land, Inner Mongolia, China (Fig. 4). This region has a continental semiarid monsoon temperate climate that is characterized by a dry and windy winter and spring, a warm and comparatively wet summer, and a short, cool autumn. The mean annual temperature is 6.8 °C, and the mean monthly temperatures range from a minimum of −13.2 °C in January to a maximum of 23.5 °C in July. The mean annual precipitation is 366 mm, with 70% occurring from June to August. The mean wind speed is 4.3 m/s, with occasional occurrences of gales ≥20 m/s in winter and spring, when the vegetation cover is lowest and the soil is driest^46^.

## Methods

### Experimental design

A space-for-time substitution approach^47^ was used in the present work. Specifically, 3 sites, representing three main stages of dune stabilization (mobile dunes, semi-fixed dunes and fixed dunes), were selected in close proximity to each other (maximum distance of 5 km) near the Naiman Desertification Research Station. The experiments at the three sites used a randomized split-plot design with four replications (Fig. 4). The semi-fixed and fixed dunes selected for this study were mobile dunes that were naturally restored using fencing since 2012 and 1999, respectively. These sites have not been grazed since the time of enclosure. All sites were initially mobile dunes caused by long-term overgrazing. In Naiman Banner, livestock grazing has been strictly prohibited from April to June since 1999. Therefore, the *Caragana* shrubs in the mobile dunes were not available to grazing animals during our experimental period.

Soil property measurements showed that soil nutrients at the soil surface increased significantly with restoration (Table S1). A vegetation survey was performed in August 2015, at the peak of vegetation cover and species richness. The results showed that plant species richness and vegetation cover increased significantly with restoration (Figure S1).

### Pollinator observations

We identified bee species and recorded the flower-visiting rates of bee pollinators to *C. microphylla* flowers from the mobile to the fixed dune populations in 2014 and 2015. Two observers recorded the flower visitation rates by bee pollinators at least once on sunny, non-windy days at the peak of flowering over the two-year period. The pollinator visitation rates were estimated by watching 6 plants for 20-minute intervals from ~07:30 to 17:30 on each observation day and recording the number of flower visits by bee pollinators. In total, 288 observations (180 in 2014 and 108 in 2015) with each observation period lasting 20 minutes, were made of visitation rates over both years. We could not reliably identify the bee species in the field. Therefore, specimens of each flower-visiting bee species were collected and identified. The bee species richness was estimated by the total number of bee species in the focal populations. Pollinator assemblage was defined as all bee species that pollinated flower. Abundance of pollinator species was expressed as the bee pollinator visitation rate (calculated as visits per flower per hour). The relative frequency of visits by each bee visitor was calculated as the percentage of visits in each dune type.

### Experimental determination of pollen limitation

To estimate the variation in pollen limitation during vegetation restoration, we conducted a pollen supplementation experiment. At each site, we randomly selected 7–21 shrubs in May. Each plant was subjected to two pollination treatments: (1) natural pollination (Control) in which the flowers were pollinated naturally and (2) supplemental outcross treatment (PA) in which the flowers were hand pollinated with outcross pollen. Each treatment was replicated on 14–31 flowers per plant, distributed across 1–2 branches per treatment. The study sites were visited every other day. On each visit, all open flowers on the branches selected for PA were pollinated by hand with cross pollen from *C. microphylla* individuals located 30 m away and bagged. Because the flowers remained open for approximately five days, this protocol ensured that all flowers received supplemental pollen at least once. The flowers used for PA were recently opened (identified by the morphology of the anthers and flower) and were hand pollinated between 9:00 and 15:30.

In early July 2014 and late June 2015, the number of experimental flowers that had produced fruits was counted to obtain the fruit set for each plant and treatment. After the fruits had matured, the ripe fruits were collected and dissected, and then the number of seeds per fruit was counted. For each individual plant, the fruit set and mean number of seeds per fruit in each treatment were used to calculate a PL index, expressed as PL = 1 − C/PA, where C and PA represent the fruit set or mean number of seeds per fruit in the unmanipulated and manipulated treatments, respectively^48,49^. The pollen limitation index of the population was calculated by averaging the individual PL values. This standard index ranges from 0 (indicating no pollen limitation) to 1 (indicating the highest pollen limitation). The cumulative pollen limitation index, calculated as PL_cumulative_ = 1 − (C_fruit set_ C_seeds per fruit_)/(PA_fruit set_ PA_seeds per fruit_), was used to examine the pollen limitation for seed production in each individual^49^.

### Data analysis

The results are expressed as the mean ± standard error (SE). The effects of vegetation restoration and year on the visitation rate and the PL were tested using the General Linear Model (GLM) for a split-plot design. A simple linear regression was performed between the mean visitation rates and richness of bee pollinators and the mean PL of each replication in the three studied sites during two years to examine whether variations in visitation rate affected the intensity of PL. The analyses were performed using SPSS 16.0, and all *P*-values were considered significant at the 0.05 level.

## Electronic supplementary material

Supplemental material

## References

[1] Schwilch G, Bachmann F, Liniger HP (2009). Appraising and selecting conservation measures to mitigate desertification and land degradation based on stakeholder participation and global best practices. Land Degradation and Development..

[2] Zhao, H. L., Zhao, X. Y., Zhang, T. H. & Zhou, R. L. Bioprocess of desertification and restoration mechanism of degraded vegetation. Science Press, Beijing (2007).

[3] Liu RT, Zhao HL, Zhao XY (2009). Effect of vegetation restoration on ant nest-building activities following dune stabilization in Horqin Sandy Land, North China. Land Degradation and Development..

[4] Zuo XA (2009). Spatial heterogeneity of soil properties and vegetation–soil relationships following vegetation restoration of mobile dunes in Horqin Sandy Land, Northern China. Plant and Soil..

[5] Li YQ (2013). *Accumulation of carbon and nitrogen in* the plant–soil system after afforestation of active sand dunes in China’s Horqin Sandy Land. Agriculture, Ecosystems & Environment..

[6] Lomov B, Keith DA, Hochuli DF (2009). Linking ecological function to species composition in ecological restoration: seed removal by ants in recreated woodland. Austral Ecology..

[7] Cortina J (2006). Ecosystem structure, function, and restoration success: Are they related?. Journal for Nature Conservation..

[8] Ollerton J, Winfree R, Tarrant S (2011). How many flowering plants are pollinated by animals?. Oikos..

[9] Kearns CA, Inouye DW, Waser NM (1998). Endangered mutualisms: the conservation of plant-pollinator interactions. Annual Review of Ecology and Systematics..

[10] Kremen C, Ricketts T (2000). Global perspectives on pollination disruptions. Conservation Biology..

[11] Hopwood JL (2008). The contribution of roadside grassland restorations to native bee conservation. Biological conservation..

[12] Morandin LA, Kremen C (2013). Hedgerow restoration promotes pollinator populations and exports native bees to adjacent fields. Ecological Application..

[13] Forup ML, Henson KSE, Craze PG, Memmott J (2008). The restoration of ecological interactions: plant–pollinator networks on ancient and restored heathlands. Journal of Applied Ecology..

[14] Lomov B, Keith DA, Hochuli DF (2010). Pollination and plant reproductive success in restored urban landscapes dominated by a pervasive exotic pollinator. Landscape and Urban Planning..

[15] Reed MS (2013). Knowledge management for land degradation monitoring and assessment: an analysis of contemporary thinking. Land Degradation and Development..

[16] Zhu, Z. D. & Chen, G. The sandy desertification in China, vol. 7, Science Press, Beijing (1994).

[17] Zuo XA (2008). Plant distribution at the mobile dune scale and its relevance to soil properties and topographic features. Environmental Geology..

[18] Zhao HL, Zhou RL, Zhao XY, Zhang TH (2008). Ground discriminance on positive and negative processes of land desertification in Horqin Sand Land. Journal of Desert Research..

[19] Zhao YZ (2005). The distribution pattern and ecological adaptation of *Caragana mirophylla*, *Caragana davazamcii* and *Caragana korshinskii*. Acta Ecologica Sinica.

[20] Knight TM (2005). Pollen limitation of plant reproduction: pattern and process. Annual Review of Ecology and Systematics..

[21] Odum EP (1969). The strategy of ecosystem development. Science..

[22] Brown, V. K. & Southwood, T. R. E. Secondary succession: patterns and strategies. In Colonization, succession and stability, Gray AJ, Crawley MJ, Edwards DJ (eds). Blackwell; 315–337 (1987).

[23] Brown VK, Hyman PS (1986). Successional communities of plants and phytophagous Coleoptera. Journal of Ecology..

[24] Steffan-Dewenter I, Tscharntke T (2001). Succession of bee communities on fallows. Ecography..

[25] Potts SG (2003). Linking bees and flowers: how do floral communities structure pollinator communities?. Ecology..

[26] Ghazoul J (2006). Floral diversity and the facilitation of pollination. Journal of Ecology..

[27] Westrich, P. The Conservation of Bees. Academic Press for the Linnean Society of London and IBRA, London (2006).

[28] Alford DV (1969). A study of the hibernation of bumblebees (Hymenoptera: Bombidae) in Southern England. Journal of Animal Ecology..

[29] Goulson, D. Bumblebees: their behaviour and ecology. Oxford University Press, Oxford, United Kingdom (2003).

[30] Westphal C, Steffan-Dewenter I, Tscharntke T (2003). Mass flowering crops enhance pollinator densities at a landscape scale. Ecology Letters..

[31] Burd M (1994). Bateman’s principle and plant reproduction: the role of pollen limitation in fruit and seed set. Botanical Reviews..

[32] Eriksson O, Jakobsson A (1998). Abundance, distribution and life histories of grassland plants: a comparative study of 81 species. Journal of Ecology..

[33] Hill LM, Brody AK, Tedesco CL (2008). Mating strategies and pollen limitation in a globally threatened perennial *Polemonium vanbruntiae*. Acta Oecologica..

[34] Fernández JD, Bosch J, Nieto-Ariza B, Gómez JM (2012). Pollen limitation in a narrow endemic plant: geographical variation and driving factors. Oecologia..

[35] Gómez JM (2010). Changes in pollinator fauna cause spatial variation in pollen limitation. Journal of Ecology..

[36] Cusser S, Neff JL, Jha S (2016). Natural land cover drives pollinator abundance and richness, leading to reductions in pollen limitation in cotton agroecosystems. Agriculture, Ecosystems and Environment..

[37] Peterson, G., Allen, C. R. & Holling, C. S. Ecological resilience, biodiversity, and scale. *Ecosystems* **11**, 6–18 (1998).

[38] Hoehn, P., Tscharntke, T., Tylianakis, J. M. & Steffan-Dewenter, I. Functional group diversity of bee pollinators increases cropyield. *Proc. R. Soc. B.* **275**, 2283–2291 (2008). 10.1098/rspb.2008.0405 PMC2603237 18595841

[39] Klein AM, Steffan-Dewenter J, Tscharntke T (2003). Fruit set of highland coffee increases with the diversity of pollinating bees. Proceedings of the Royal Society B..

[40] Albrecht M, Schmid B, Hautier Y, Müller CB (2012). Diverse pollinator communities enhance plant reproductive success. Proceedings of the Royal Society B..

[41] Fussell M, Corbet SA (1992). Flower usage by bumblebees: a basis for forage plant management. Journal of Applied Ecology..

[42] Corbet SA (1995). The competition box: a graphical aid to forecasting pollinator performance. Journal of Applied Ecology..

[43] Fontaine, C., Dajoz, J., Meriguet, J. & Loreau, M. Functional diversity of plant-pollinator interaction webs enhances the persistence of plant communities. *PLoS biology* **4**, 129–135 (2005). 10.1371/journal.pbio.0040001 PMC1310649 16332160

[44] Karamysheva, Z. V. & Khamtsov, V. N. The steppe of Mongolia (Review of Geobotanical Monographs). Braun-Blanquetia volume. 17. Centro Audiovisivi e Stempa’Publisher, Camerino (1995).

[45] Narantsetseg A, Kang S, Lkhamsuren BE, Ko DW (2014). Determinants of *Caragana microphylla* density distribution in the Mongolian steppe. Ecological research..

[46] Li YQ (2012). Mongolian pine plantations improve soil physico-chemical properties and enhance soil carbon and nitrogen capacities in semiarid sandy land. Applied Soil Ecology..

[47] Pickett, S. T. A. Space-for-time substitution of as an alternative to long-term studies. In Long-term studies in ecology: approaches and alternatives, Likens GE (ed). Springer, New York; 110–135 (1989).

[48] Larson BMH, Barrett SCH (2000). A comparative analysis of pollen limitation in flowering plants. Biological Journal of the Linnean Society..

[49] González-Varo JP, Arroyo J, Aparicio A (2009). Effects of fragmentation on pollinator assemblage, pollen limitation and seed production of Mediterranean myrtle (Myrtus communis). Biological Conservation..

