# Supplementary material for: Increased pollinator service and reduced pollen limitation in the fixed dune populations of a desert shrub
Source: Sci Rep. 2017 Dec 4;7:16903. doi: 10.1038/s41598-017-17253-w (PMC5715164; doi:10.1038/s41598-017-17253-w)
Supplement: Supplementary file 1 — Supplemental material [file 41598_2017_17253_MOESM1_ESM.doc]

**Increased pollinator service and** **reduced pollen limitation** **in the fixed dune populations of a desert shrub**

Cheng-Chen Pan^1,2^,*, Hao Qu^1^,*, Qi Feng^1,2^, Lin-De Liu^3^, Ha-Lin Zhao^1^, Yu-Lin Li^1^, Yu-Qiang Li^1^, Tong-Hui Zhang^1^ & Xin-PingLiu^1^

^1^*Northwest Institute of Eco-Environment and Resources,* *Chinese Academy of Sciences, Lanzhou 730000, China*

^2^*Key Laboratory of Ecohydrology of Inland River Basin, Chinese Academy of Sciences, Lanzhou 730000, China*

^3^*College of Life Sciences,* *Ludong University, Yantai 264025, China*

∗These authors are co-first authors and contributed equally to this work. Correspondence and requests for materials should be addressed to Q. Feng (email: qifeng@lzb.ac.cn) or L.D. Liu (email: linde_liu@163.com)


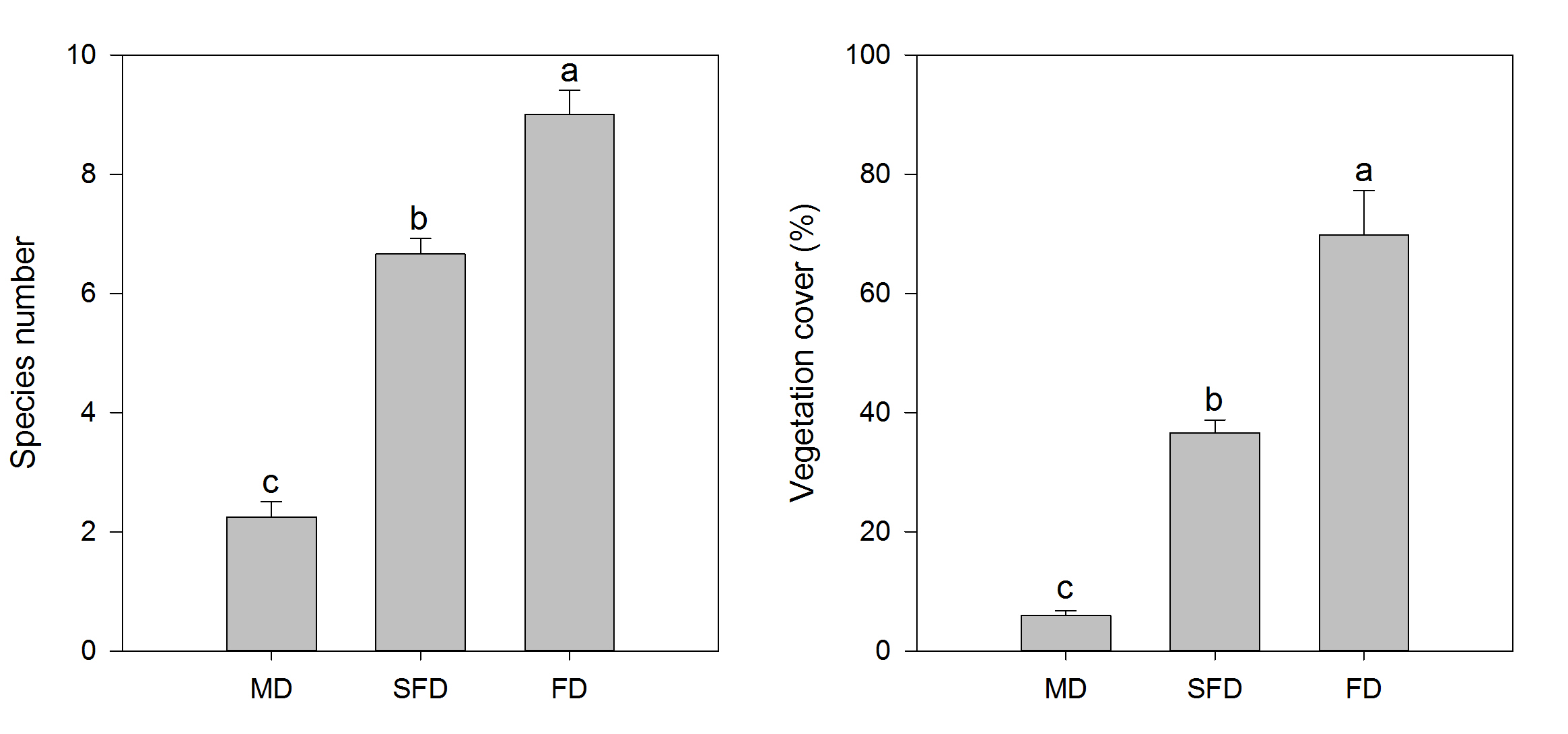


**Figure S1** Changes in vegetation characteristics at the different restoration stages in 2015. Values (±SE) are means of all sites for mobile dunes (MD), semi-fixed dunes (SFD) and fixed dunes (FD). Different letters indicate significant differences among dune types (*P* < 0.05)

**Table S1 Changes in soil chemical properties (0-40cm) at the different restoration stages. Values (±SE) are means of all sites for** **mobile dunes (MD), semi-fixed dunes (SFD) and fixed dunes (FD).**

|  | Depth (cm ) | SOC (g/kg) | TN (g/kg) | TP (g/kg ) | AN (mg/kg) | AP (mg/kg) |
| --- | --- | --- | --- | --- | --- | --- |
| MD | 0-10 | 0.27±0.01 | 0.06±0.01 | 0.06±0.00 | 21.5±2.2 | 4.7±0.1 |
|  | 10-20 | 0.31±0.05 | 0.09±0.01 | 0.06±0.00 | 22.6±0.8 | 4.9±0.5 |
|  | 20-40 | 0.32±0.01 | 0.07±0.01 | 0.05±0.01 | 24.1±1.0 | 5.0±0.3 |
| SFD | 0-10 | 1.03±0.28 | 0.16±0.01 | 0.08±0.01 | 24.4±4.0 | 7.4±1.1 |
|  | 10-20 | 0.63±0.48 | 0.10±0.01 | 0.05±0.01 | 27.7±1.1 | 6.2±0.9 |
|  | 20-40 | 0.39±0.02 | 0.08±0.02 | 0.05±0.00 | 24.1±1.0 | 2.5±0.1 |
| FD | 0-10 | 2.99±0.57 | 0.36±0.03 | 0.12±0.01 | 46.1±4.4 | 11.4±0.8 |
|  | 10-20 | 1.18±0.26 | 0.20±0.03 | 0.08±0.00 | 29.8±0.5 | 4.6±0.8 |
|  | 20-40 | 0.57±0.06 | 0.09±0.01 | 0.51±0.00 | 22.6±2.4 | 2.6±0.2 |

**Table S2** The species of flowering plants utilization by bees in mobile dunes (MD), semi-fixed dunes (SFD) and fixed dunes (FD) in 2015

|  | MD | SFD | FD |
| --- | --- | --- | --- |
| *Caragana microphylla* | √ | √ | √ |
| *Lightyellow Sophora* |  |  | √ |
| *Lespedeza davurica* |  |  | √ |
| *Leonurus artemisia* |  |  | √ |
| *Hedysarum fruticosum* |  |  | √ |
| *Vicia lilacina* |  |  | √ |
